# Road Transport of Farm Animals: Mortality, Morbidity, Species and Country of Origin at a Southern Italian Control Post

**DOI:** 10.3390/ani8090155

**Published:** 2018-09-17

**Authors:** Barbara Padalino, Daniele Tullio, Saverio Cannone, Giancarlo Bozzo

**Affiliations:** 1 Department of Veterinary Medicine, University of Bari, 70010 Bari, Italy; s.cannone8@studenti.uniba.it (S.C.); giancarlo.bozzo@uniba.it (G.B.); 2 Jockey Club College of Veterinary Medicine and Life Sciences, City University of Hong Kong, Kowloon, Hong Kong, China; 3 ASL BA—Local Health Authority Veterinary Service, 70100 Bari, Italy; daniele.tullio@asl.bari.it

**Keywords:** livestock, transport, control post, health, welfare

## Abstract

**Simple Summary:**

Long distance transportation is a welfare concern because it may cause sickness (i.e., morbidity) or death (i.e., mortality). Commercial transportation in Europe is regulated by the Council Regulation (EC) No. 1/2005 which regulates the maximum journey in the different species. After this time animals must be unloaded for resting, watering and feeding at control posts (CPs) where Official Veterinarians (OVs) have to check their health. This study analyzed the surveillance reports filled by OVs at a CP in Southern Italy from 2010 to 2015. A total of 1391 trucks stopped at the CP, transporting a total of 111,536 animals. The average mortality and morbidity rates were 0.025% and 0.010%. Cases of mortality and/or morbidity were reported for only 11 out of the 1391 trucks (0.8%). In a truck transporting lambs, 14 dead on arrival (DOA) were recorded, and this represented 93% of all DOAs. This is the first study reporting the results of surveillance practices conducted by OVs on animals travelling from North Europe to a CP in Southern Italy in compliance with EC 1/2005. Further studies should be conducted comparing the implications of long distance transportation at different CPs along different routes.

**Abstract:**

Statistics on animal transport and its implications for health and welfare are limited. This study documented the animals transiting through a control post and their welfare outcomes measured by mortality rate and the prevalence of animals considered unfit for further transport (i.e., morbidity). Reports filed by the director of the control post and Official Veterinarians from 2010 to 2015 were analyzed. A total of 60,454 (54.2%) sheep/goats, 45,749 (41.0%) cattle, and 5333 (4.8%) pigs travelled in 225 (16.2%), 1116 (80.2%) and 50 (3.6%) trucks, respectively. Trucks coming mainly from France (71.3%), Spain (14.0%), and Ireland (7.4%) went mainly to Greece (95.4%), which was also the most common nationality of the transport companies (44.6%). Cases of mortality and/or morbidity were reported for only 11 out of the 1391 trucks (0.8%). The average mortality and morbidity rates were 0.025% and 0.010%, with maximum values for transport of lambs (0.084%, and 0.019%). Species of animal being transported and space allowance were associated with the measured welfare outcomes (*p* < 0.05). Overall, this study provided statistics based on official surveillance reports, suggesting that small space allowance during long haul transportation of sheep/goats may affect their health and welfare.

## 1. Introduction

Millions of animals are transported daily all over the world. The movement of livestock across the borders of Member States of the European Union is monitored using the Trade Control and Expert System (TRACES) and reported in the Activity Report [1]. For example, approximately 3 million head of cattle are transported for fattening annually. However, long distance animal transport is an animal welfare issue, because it is a stressfull event triggering often the onset of health problems [2]. With the aim of reducing transport stress and consequently the incidence of transport-related health and welfare issues, many studies have been published identifying risk factors for farm animals pre-, during and post-road transport [3]. Pre-journey risk factors include many factors, such as on-farm handling, rearing conditions, assembly of animals, classifying, weighing, repenning in a new environment, re-grouping, mixing with unfamiliar animals, fitness for transport and handling at loading [4,5]. Among risk factors during the journey are journey duration, withdrawal of feed and water, thermal and physical conditions inside the vehicle, overcrowding, absence of partitions, driving skills, noise, vibration, and road quality [6,7,8]. Post-journey risk factors include handling at unloading, duration of rest period, recovery practices, re-grouping, and mixing with unfamiliar animals [9,10,11,12].

One of the determining risk factors is journey duration [13,14]. Consequently, Council Regulation (EC) No. 1/2005, which regulates animal transport in Europe, includes special requirements for journeys exceeding 8 h. For instance, maximum journey duration is 29 h for ruminants and 24 h for horses and pigs. After this time animals must be unloaded for resting, watering and feeding for at least 24 h in locations approved by the competent authorities [15]. Such locations used to be called staging points in Council Regulation (EC) No. 1255/1997 and have now been renamed control posts (CPs) by Council Regulation (EC) No. 1/2005. Council Regulation (EC) No. 1255/1997 (Article 6) requires that official veterinarians (OVs) inspect the means of transport and accompanying documents, as well as evaluate the animals’ fitness for transport before the animals leave the control post again. The facilities and management at CPs have been identified as key factors in animal recovery, affecting both resting behaviour and biochemical parameters [10,11,15]. However, scientific literature regarding the effect of CPs on animal welfare during long distance road transports is still limited.

Surveys on farm animal transport have been performed to explore the epidemiological basis of transport-related health and welfare issues worldwide. For instance, the mortality due to road transport has been calculated for beef cattle in North America (0.01%) [16], fattening pigs in Europe (0.07%) [17], and bobby calves in Australia (0.64%) [13].Whilst death is a definitive welfare outcome, the variation in the above mentioned mortality is most likely related to the species or the type of animals being transported and their transport and handling conditions [18], or else to the journey duration [14]. The prevalence of transport-related health problems varied significantly even within the same species (e.g., in slaughter horses injury rate varied from 7% to 28% [19,20]). One reason for this large variation may be the use of different criteria to assess health problems. To the best of the authors’ knowledge, there is no survey reporting animal transport welfare outcomes measured by OVs at a control post. Italy currently has 13 CPs, only one of which is in Southern Italy (Doc. SANCO/2677/99 Rev.241). Consequently, the aims of this study were to document animal transits through the Southern Italian control post from 2010 to 2015 and to report the corresponding mortality and prevalence of animals considered unfit for transport using the official reports filed in compliance with Article 6 of Council Regulation (EC) No. 1255/1997 and Annexes I and II of Council Regulation (EC) No. 1/2005.

## 2. Materials and Methods

### 2.1. Dataset

With the permission of the OVs and the director of the control post, surveillance reports from 2010 to 2015 filed by the director of the control post (CP) IT CE 07/PS Bitritto (Bari, Italy) in compliance with Council Regulation (EC) No. 1255/1997 (Article 5(h)), and double-checked by OVs from the Local Health Authority (Bari, Italy) were used for this manuscript.

The CP (authorization CE 07/PS; 41°2′27″ N, 16°50′9″ E) measured approximately 8000 square metres during the data collection period and could contain up to 1200 bovines, 1850 sheep/goats, 500 pigs and 150 horses. It had a lorry wash, three hay barns, five animal houses and many different pens, equipped with watering and feeding points, bedding and resting facilities. The animal houses had adequate natural and artificial light to ensure proper inspection of the animals by the OVs.

At the control post, three OVs working for the Local Health Authority (Bari, Italy), including one of the authors (DT) checked the animals during loading, and immediately before they left the CP, to assess their fitness for transport following the criteria set out in Annex 1 of Council Regulation (EC) No. 1/2005. Depending on the day of arrival, the animals were checked by one of the three OVs. However, those three OVs had each received official EU training to assess fitness for transport and used the same check list to compile the surveillance reports. In compliance with the (EC) No. 1/2005, the OVs judged animals as unfit to continue the journey when they were either seriously injured or presented clinical signs of a pathological process, including severe lameness (animals unable to move independently without pain or to walk unassisted), prolapse, severe open wound, and respiratory and gastro-enteric disease (animals with massive nasal discharge, severe dyspnoea and pleurodynia, animals with severe diarrhoea or acute abdominal pain, respectively). Such animals were either humanely euthanized, slaughtered in the local slaughterhouse or treated depending on the severity of the case after the assessment performed by the OVs.

The following parameters were recorded in the surveillance reports: date and time of arrival, species, number of animals transported per truck (NATT), country of provenance, TRACES code, truck registration number, transport company, number of dead on arrival (DOA), of those which died at the control post (DCP), of animals judged unfit to continue travel (UFT), day and time of departure, and country of final destination. Since these reports were official documents, there were no missing data.

Based on the surveillance reports, the dates were expressed as month and season. Using the category which was reported in each TRACES, the category of transported animals was added to the dataset. The categories were chosen in compliance with Council Regulation (EC) No. 1/2005, for bovines and sheep/goats the following categories were used: small calves (100 kg), medium-sized calves (200 kg), heavy calves (325 kg), heavy cattle (550 kg), very heavy cattle (>700 kg), lambs, and sheep/goats; whereas for pigs, the following categories were applied: light fattening (110–120 kg), heavy fattening (130–150 kg), and breeding (>200 kg).

The space allowance per animal was also added to the dataset. It was calculated by dividing the available floor space in each truck by the number of animals transported in the truck and expressed as m^2^/animal. It was not possible to calculate the space allowance in kg/m^2^ because the surveillance reports did not report the total weight of the truck load. 

The transport companies were aggregated by country of origin (i.e., nationality) and the total number of dead animals was calculated as the sum of DOA and DCP.

### 2.2. Statistical Analysis 

Descriptive statistics of the data collected were obtained using an online statistical software (Statulatorbeta^®^, Sydney, Australia) with year, season, month, species, category, country of provenance, nationality of transport company and country of destination as categorical variables, while NATT, DOA, DCP, total dead, UFT and space allowance were used as numerical variables.

Chi-square tests were conducted to determine the association between factors (i.e., year, season, month, species, typology, provenance, destination). Trucks with DOA, DCP, and/or UFT were considered as having a welfare problem. A univariate logistic regression model was developed with welfare problem as a binary outcome (1/0; welfare problem/non-welfare problem) and year, season, month, species, category, provenance, destination, nationality, and space allowance as predictive variables. *P* values were calculated using the Wald test. Each predictor variable returning a *p* < 0.25 from the univariate analyses was considered for inclusion in the final multivariate model for welfare problems. Predictor variables for the final multivariate logistic regression model were selected using a step-wise backward elimination procedure, whereby predictive variables were removed until all variables in the final model had a *p* < 0.05 indicating significance [9]. The aforementioned statistical analyses were performed using GenStat^®^Version 14 (VSN International, Hemel Hempstead, UK).

The effect of year, season, month, provenance, destination, transport company nationality, species and category on NATT was determined using a General Linear Model (GLM) procedure. Tukey’s HSD (honestly significant difference) test was used as a post-hoc test. Statistical analyses were performed using SAS version 9.4. *P* threshold was set at 0.05. Data are expressed as least square means ± standard error (SE).

## 3. Results

**Figure 1:**
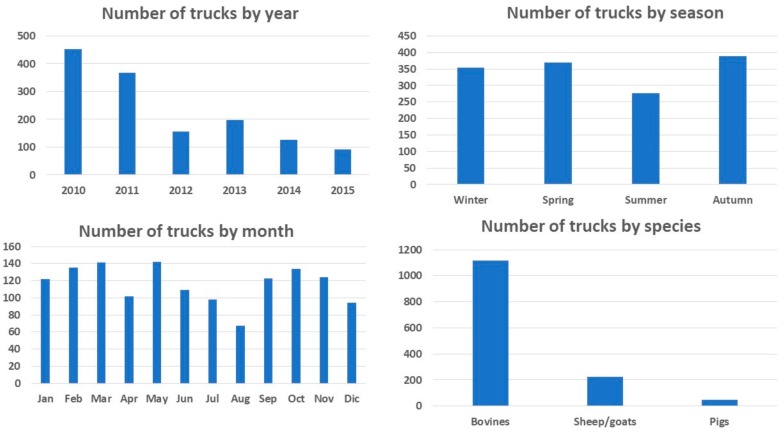
Descriptive statistical analysis of the 1391 trucks which stopped at control post CE 07/PS Bitritto (Bari, Italy) from 2010 to 2015. Number of trucks examined by categorical variables (i.e., factors) year, season, month, and species.

A total of 1391 trucks, mainly trailers and semi-trailers, stopped at the control post over the study period, transporting a total of 111,536 animals. Figure 1 shows the descriptive statistics of the categorical variables year, month, season and species. 

**Figure 2:**
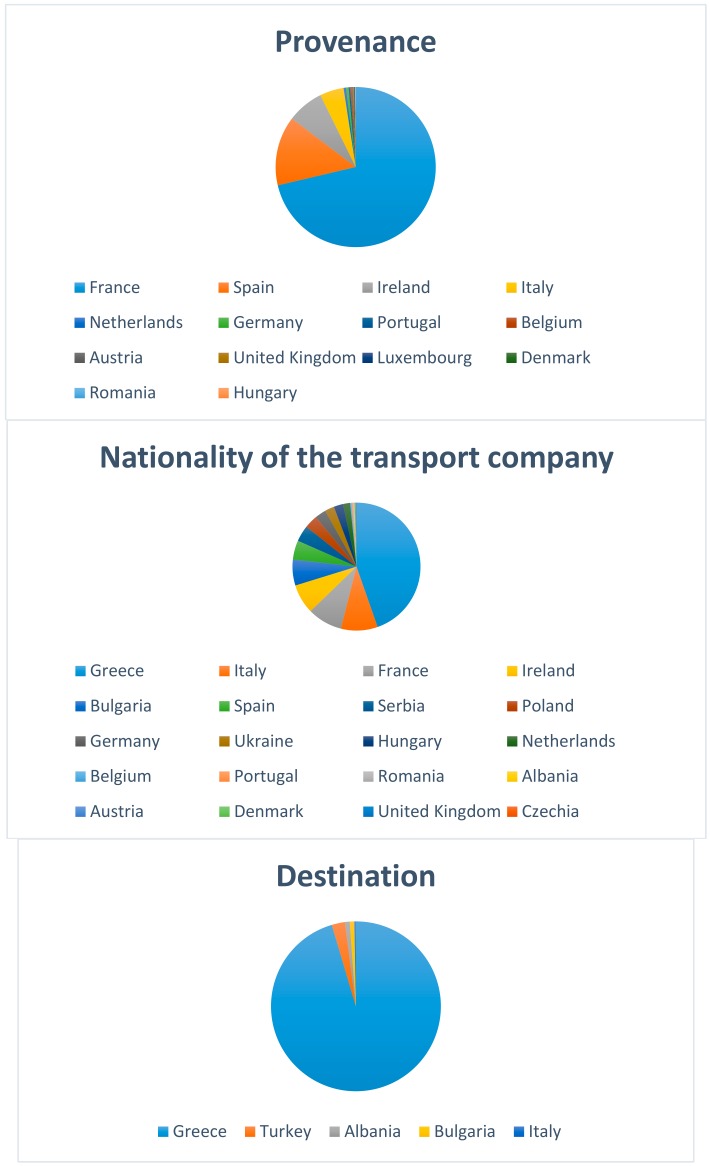
Pie chart by provenance, nationality of transport company, and destination for the 1391 trucks which stopped at control post CE 07/PS Bitritto (Bari, Italy) from 2010 to 2015.

The frequency and the percentage of all the trucks transiting across the control post based on category, provenance, nationality of transport company and destination are shown in Table 1 and Figure 2, respectively. 

The majority of the trucks transported bovines, while heavy cattle (i.e., animals with a body weight of about 550 kg) was the category most frequently transported. The most common provenances were France, Spain, and Ireland, even though the transport companies were mainly from Greece, which was also the most common final destination. There was a decrease in the number of trucks and transported animals after 2012, in particular for bovines and sheep. The transport of bovines tended to drop in August, while the transport of sheep/goats peaked in July and October (Figure S1).

Table 2 shows the descriptive statistics of the numerical variables based on the total shipments. 

The maximum number of DOAs (14) was recorded in a truck transporting a total of 214 lambs from Spain, and this represented 93% of all DOAs. 

Space allowance varied accordingly with the species, and was within the range reported in CE 1/2005, which regulates a space allowance from 0.30 to 1.60 m^2^/animal for bovines, from 0.20 to 0.50 m^2^/animal for sheep and at least 235 kg/m^2^ for pigs of 100 kg. In our study, the minimal values of space allowance resulted in lamb transportation (Median = 0.34, IQR: 0.27–0.34).

The mortality and morbidity rates were 0.025% and 0.010%, respectively, with maximum values for transport of lambs (0.084%, and 0.019%). Cases of mortality and/or morbidity were reported for only 11 out of the 1391 trucks (0.8%). Table 3 shows the number of transported animals, mortality and morbidity rates calculated in relation to the different factors studied.

**Figure 3:**
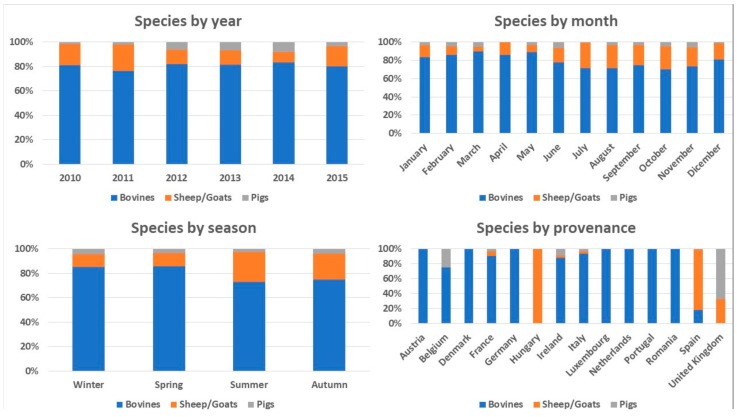
Association between species and year, month, season, provenance, nationality of transport company and destination.

There was an association between species transported and year (X^2^ = 40.25; *p* < 0.001), season (X^2^ = 38.28; *p* < 0.001), month (X^2^ = 67.19; *p* < 0.001), provenance (X^2^ = 751.88; *p* < 0.001), nationality of transport company (X^2^ = 1200.74; *p* < 0.001) and destination (X^2^ = 62.67; *p* < 0.001) (Figure 3, Figure S2).

Consequently, there was also an association between category transported and year (X^2^ = 841.29; *p* < 0.001), season (X^2^ = 90.83; *p* < 0.001), month (X^2^ = 191.79; *p* < 0.001), provenance (X^2^ = 1603.70; *p* < 0.001), nationality of transport company (X^2^ = 2114.33; *p* < 0.001) and destination (X^2^ = 192.41; *p* < 0.001). There was a higher number of trucks transporting sheep/goats in June, July and August, i.e., in summer, than in other months and seasons of the year. Sheep/goats came mainly from Spain and Hungary.

In the univariate logistic model, species of animal being transported and space allowance were the only predictive variable which proved to be associated with a welfare problem (X^2^ = 5.780; df = 2; *p* = 0.049, and X^2^ = 8.982; df = 1; *p* = 0.003, respectively) (Table S1). Trucks transporting sheep/goats were four times more likely to be associated with a case of DOA, DCP, or UFT than those transporting bovines. For a unit increase in space allowance, the odds in favor of the welfare problem occurring decreased by a factor of 0.24 (OR: 0.24; CI: 0.10–0.61) (Table 4). In the multivariate model, only space allowance remained significant (*p* < 0.05).

At the GLM, the effect of the year was not significant (*p* = 0.072) on NATT. While the number of transported animals per truck varied significantly depending on species (pigs 106.7 ± 4.7, sheep/goats 268.7 ± 2.2, cattle 41.0 ± 1.0; *p* < 0.001), the effect of the other predictive variables associated with species resulted in significant differences on NATT (Table S2).

## 4. Discussion

This study documented transport-related mortality and prevalence of animals considered unfit for transport for farm animals (i.e., cattle, sheep/goats, pigs) on a stopover at a control post in Southern Italy using reports of OVs. This study reports on the number of animals which died and were judged unfit to continue travel by OVs in compliance with Section 5 of Annex 1 of Council Regulation (EC) No. 1/2005, thus providing the first report containing statistics on farm animal movements from Northern Europe to the Balkan Peninsula. Considering that only 0.8% of the shipments was associated with one of the considered welfare issue (i.e., DOA, UFT), and that the Eurobarometer [21] reports that the perception of animal welfare has increased among European consumers, our data may help consumers gain knowledge of live animal transport within Member States.

The animals on the stopover at our CP were mainly travelling to Greece, a country which relies heavily on meat and dairy imports [22]. Animals were being transported for different purposes: for the final fattening period before slaughtering in the destination countries, for breeding, for direct sale or for slaughter. The number of vehicles stopping at the CP dropped significantly after 2012, probably due to the economic crisis in Greece [22]. The reasons for the great demand for live animal transport have been analyzed by the European Union [23] and include a high level of demand for fresh meat; use of indigenous slaughterhouse facilities; use of by-products (such as skin and offal) from the slaughter process; poor meat transport, refrigeration and storage capacity; use of religious slaughter rituals in some EU-countries. The EUR-Lex (1998) states that even though animal welfare suggests slaughtering an animal close to its origin and then transporting the carcass to its final destination, individual Member States have such conflicting interests that no consensus can be reached on the issue. Safeguarding animal welfare during transport is therefore crucial.

In our study, the majority of the trucks came from France, transporting mainly bovines. This was expected, since France exports millions of stocker calves to other EU countries each year for fattening [7]. Spain was the second most common provenance, but these vehicles were transporting sheep/goats; this was also expected, since Spain has the second-highest number of sheep (a total of 18,136,050) amongst European Union Member States [24]. The transport companies were mainly Greek, probably because the final destination was Greece. Indeed, Greece has the highest per capita consumption of sheep meat in Europe and is a major importer of sheep [25]. However, such data lead us to reflect on the importance of live animal transport for the Greek economy and of the training of Greek livestock hauliers. Europe is currently the only part of the world where hauliers are required to undergo training, but according to a 2007 survey, only 33.3% of the hauliers had attended training courses provided either by the Local Health Authority or trade organizations [26]. More recently, a survey proved the need of additional education and training for livestock drivers in Denmark [27]. Consequently, courses for transporters should be implemented and promoted worldwide, since training for all those involved in live animal transport has been identified as a key factor for improving welfare outcomes among transported animals [28].

Mortality is often used as an animal welfare indicator, since it is beyond doubt that such deaths are preceded by a period of suffering and poor welfare [14]. In relation to transport, mortality has often been reported as DOA, down on trailer or before weighing and total dead [29]. In our case, DOA, DCP and total dead showed an overall mortality rate within the range reported in the literature [25]. In our study, the mortality rate for bovines was similar to the rates reported in Canada (0.011%) [16] and in Czechia (0.012%) [30]. Contrastingly, the mortality for sheep/goats was outside the range (0.006–0.018%) reported in literature [31]. The highest mortality rate was found in lambs, probably because they are more likely to suffer from protracted transport stress than adult sheep and because they are more likely to be transported in large loads [31].

The mortality in pigs was 0%, lower than reported by Averós et al. in 2010 [17]. However, the latter authors focused on weaned piglets, while in our study fattened or breeding pigs were being transported, so they were older and often being transported in single compartments. All these factors may have helped the animals cope better with the long journey, thus reducing the overall mortality rate [14]. However, the number of pig transport records in this study is low, so this should also be taken into account when interpreting our data.

The morbidity rate was lower than reported in literature [2,16,29] and the reasons for this discrepancy may be due to a variety of factors. Firstly, the criteria for assessing morbidity in the above mentioned studies varied from one author to the next, while our reports were filed by OVs based on the criteria set out in Council Regulation (EC) No. 1/2005 for the fitness for transport. The OVs stopped only those animals showing severe injuries and diseases; consequently, no minor injuries or pathologies were counted in our dataset, thus reducing the morbidity rate. Secondly, the enforcement of a correct assessment of fitness for transport pre-departure may have played a role in reducing morbidity. While Marlin et al. (2011) reported that many transport related injuries assessed at unloading were due to animals’ pre-existing poor health and welfare status, the number of animals stopped at departure was not considered in our dataset. Finally, the majority of publications assess morbidity at the slaughterhouse, while our data are the first to record it at a CP; our animals could have had time to recover from the long journey at the CP, being milked and humanely handled by the technicians working there. It has recently been shown that sheep rested for at least 16 h at a CP recovered from the stress, dehydration and fatigue induced by a 29-h journey [11]. The goal of CPs is indeed to give the animals the time to recover from the effects of a long journey before being loaded and transported again. Unfortunately, there are very few scientific studies on the effects of different management systems and resting period at CPs [10,11]. Our data could not therefore compared with the literature. Further studies should be conducted comparing transport related morbidity at different CPs and in more countries.

In this study, sheep/goats were found to be more likely affected by transport than the other types of animals. It has been reported that sheep usually cope better with transport than other species [31], though there have been occasional reports of high mortality among single loads of animals during journeys that are non-compliant with regulations, such as an infamous case of 65 deaths out of 400 sheep being transported from Poland to France [31]. Similarly, our rate may have been affected by the fact that 14 out of 214 lambs were found dead on arrival in a single vehicle, which proved to be not compliant with EU regulation on space allowance and fined for overcrowding. It has been assumed that transport is less tiring for sheep/goats because as opposed to cattle and horses, both lambs and adult sheep lie down during transport [14]. However, studies have documented that transport reduced resting and rumination behaviours of lambs which showed signs of stress soon after loading and high levels of dehydration and weight loss after journeys lasting 12 h [31,32]. The number of inspections should be increased on trucks transporting lambs in Europe.

Overcrowding has been identified as a risk factor for transport-related health and welfare issues and it was the most frequent infringement observed during vehicle inspections carried out between 2001 and 2010 at the border between France and Italy [7]. Council Regulation (EC) No. 1/2005 gives a stocking density range for bovines and sheep/goats expressed in m^2^/animal, which vary according to the category of animal transported. The range for lambs goes from 0.20 to 0.30 m^2^/animal. However, it has been shown that stocking density should not be expressed in square meters per animal but in square meters per 100 kg to allow lambs to lie down and cope better with transport stress, and that lamb health and welfare may be affected by high stocking density [32]. Our data may be useful to implement the existing European Transport Guidelines and further studies should be performed to determine the optimal stocking density of lambs transported over long journeys within Europe.

Journey duration was confirmed as the most important risk factor in the development of transport-related diseases [33]. Surprisingly, in our data set there was no association between welfare outcomes and provenance. One reason for this finding may be related to the fact that the journeys had to follow a fixed route which was checked and approved by OVs before departure. However, it may also be due to the fact that we could not ascertain the exact journey duration in our dataset and that we used provenance as a predictive variable. Further studies should be conducted on a larger dataset using journey duration as a predictive variable to ascertain our findings.

Season and month have been identified as risk factors for transport-related mortality [29,30,33]. However, in our study, neither season nor month were significant. Our results are similar to those reported for pig transport by Gosálvez et al. 2006 [34], who explained their results by Spanish pig hauliers taking precautions to protect animals from extreme conditions, such as undertaking journeys at night, reducing loading densities and showering animals. Apart from those precautions taken by the hauliers, our results might also be due to the development of new vehicles. Those vehicle must have appropriated ventilation systems, temperature monitoring and recording systems [35], to comply with the new standards required by Council Regulation (EC) No. 1/2005 for journeys exceeding 8 h. These new technologies may have enhanced the welfare of the transported animals, assuring thermal comfort throughout the year. Overall, our data should be interpreted with caution and confirmed using a larger dataset.

The NATT was affected by many of the factors studied, in particular by species and category. There was a high average number of transported animals among vehicles transporting sheep/goats or younger—and consequently less heavy—animals. Since species and category were associated with other factors such as provenance, nationality of the transport company and destination, the effects of these latter factors on the number of transported animals per truck should be interpreted as a consequence. For instance, since sheep/goats came mainly from Spain and Hungary, all trucks coming from those countries contained higher than average numbers of transported animals. The association between species and category with season or month could, however, reflect consumer trends (e.g., eating more meat in winter; traditional consumption of lamb during religious events) and the typical commercial life of each animal. For instance, lambs are usually born in spring, weaned after three months and are then shipped to feed lot or slaughter; this could explain why we found that the transport of sheep/goats was positively associated with summer months.

Our data should be considered preliminary because this study was limited by a number of factors. Firstly, due to the small number of events (welfare problem occurred) the logistic model was likely to suffer from small-sample bias. Secondly, the surveillance reports were cumulative by truck, which made it impossible to perform a proper risk analysis based on single transported animals. Thirdly, as the assessment of welfare outcomes was related only to death and severe pathology, in compliance with Section 5 of Annex 1 to Council Regulation (EC) No. 1/2005, many minor injuries and pathologies were not recorded and consequently were not analyzed in this manuscript. Finally, as previously discussed, our dataset was unable to include the health condition of the animals before the journey, the exact duration of the journey and stocking density expressed in m^2^/100 kg. Notwithstanding these limitations, this is the first paper reporting statistics based on surveillance reports filed by official veterinarians for livestock transported from Northern Europe to the Balkan Peninsula and transiting through a control post in Southern Italy.

## 5. Conclusions

This was the first study documenting farm animal movements from Northern Europe to Greece transiting through a control post and their subsequent welfare outcomes as assessed by OVs. Only 0.8% of the trucks reported a case of mortality or morbidity and lambs transported with minimal space allowance proved to be at higher risk of poor welfare. Due to the aforementioned limitations, our findings should be considered preliminary. Further studies should be carried out in more CPs and in other countries to confirm our data. Further studies should also assess welfare on individual transported animals to identify risk factors and to analyze welfare outcomes among transported animals, comparing the welfare assessment based on official reports versus the scientific welfare assessments suggested in more recent literature.

## Figures and Tables

**Table 1:** Frequency table by category of animal transported for the 1391 trucks which stopped at control post CE 07/PS Bitritto (Bari, Italy) from 2010 to 2015.

Species	Category	Frequency	Percentage
Sheep/goats	Sheep/goats	161	11.6
	Lambs	64	4.6
	Total	225	16.2
Bovines	Medium size calves (100 kg)	136	9.8
	Heavy calves (200 kg)	165	11.9
	Medium size cattle (325 kg)	96	6.9
	Heavy cattle (550 kg)	708	50.9
	Very heavy cattle (>700 kg)	11	0.8
	Total	1116	80.2
Pigs	Light fattening (100–120 kg)	40	2.8
	Heavy fattening (130–150 kg)	5	0.4
	Breeding	5	0.4
	Total	50	3.6
Total		1391	100

**Table 2:** Descriptive statistics of the numerical variables for the 1391 trucks which stopped at control post CE 07/PS Bitritto (Bari, Italy) from 2010 to 2015.

Variable	Mean	SD	Q1	Median	Q3	Minimum	Maximum	Sum of Values
NATT	80.18	90.20	32	37	65	9	505	111536
DOA	0.01	0.38	0	0	0	0	14	15
DCP	0.01	0.15	0	0	0	0	4	13
Total dead	0.02	0.40	0	0	0	0	14	28
UFT	0.01	0.15	0	0	0	0	4	12
Space allowance	1.58	0.75	1.06	1.84	2.13	0.21	7.56	.

**Legend.** SD: Standard Deviation; Q1: first quartile; Q3: third quartile; NATT: number of animals transported per truck; DOA: Dead on Arrival; DCP: Dead at the Control Post; UFT: Unfit for Continue to Travel.

**Table 3:** Frequency table with overall calculated mortality and morbidity rates and calculated mortality and morbidity rates per year, month, season, species, and category. Total dead = dead on arrival (DOA) + dead at the control post (DCP).

Factor	Animals (n)	Total Dead (n)	Mortality Rate (%)	UFT (n)	Morbidity Rate (%)
Year					
2010	34,032	4	0.011%	4	0.011%
2011	30,171	5	0.016%	5	0.016%
2012	12,151	16	0.131%	1	0.008%
2013	16,427	2	0.012%	0	0
2014	9146	0	0.000%	1	0.010%
2015	9609	1	0.010%	1	0.010%
**Month**					
January	8339	1	0.011%	0	0
February	8685	4	0.046%	4	0.045%
March	8064	0	0.000%	0	0
April	8240	1	0.012%	1	0.012%
May	8205	0	0.000%	1	0.012%
June	9421	0	0.000%	0	0
July	10,013	2	0.019%	1	0.009%
August	6598	1	0.015%	1	0.015%
September	11,040	14	0.126%	0	0
October	13,776	4	0.029%	4	0.029%
November	11,413	1	0.008%	0	0
December	7742	0	0.000%	0	0
**Season**					
Winter	23,169	5	0.021%	4	0.017%
Spring	26,117	1	0.003%	2	0.007%
Summer	26,697	3	0.011%	2	0.007%
Autumn	35,553	19	0.053%	4	0.011%
**Species**					
Pigs	5333	0	0.000%	0	0
Sheep/goats	60,454	23	0.038%	9	0.014%
Bovines	45,749	5	0.010%	3	0.006%
**Category**					
Sheep/goats	39,028	5	0.012%	5	0.012%
Lambs	21,426	18	0.084%	4	0.019%
Medium size calves	9177	1	0.011%	1	0.011%
Heavy calves	9136	4	0.044%	1	0.011%
Medium size cattle	4188	0	0.000%	0	0%
Heavy cattle	23,009	0	0.000%	1	0.004%
Very heavy cattle	239	0	0.000%	0	0%
Light fattening	4758	0	0.000%	0	0%
Heavy fattening	350	0	0.000%	0	0%
Breeding	225	0	0.000%	0	0%

**Legend.** UFT: Unfit to Continue Travel.

**Table 4:** Results of the univariate regression analysis between welfare problem (trucks reported with a case of DOA, DCP or UFT) and species of the animal being transported. Data were collected from trucks (n = 1391) transiting across a control post in Southern Italy from 2010 to 2015.

Variable	Category	Estimate	SE	OR	95% CI	*p*
Space Allowance		−1.400	0.469	0.24	0.10–0.61	0.003
Species	Bovines	Ref		Ref		
	Sheep/goats	1.437	0.610	4.20	1.27–13.91	0.018
	Pigs	−4.3	10.3	0.01	0–69,665	0.672

**Legend.** SE: standar error; OR: odds ratio; CI: confidence interval.

## Supplementary Materials

The following are available online at http://www.mdpi.com/2076-2615/8/9/155/s1, Figure S1: Trends of the number of trucks by year and species and by month and species, Figure S2: Association between species and nationality of Transport Company and destination, Table S1: Wald test *p*-values generated from univariate regression analysis, Table S2: Results of the GLM with year, month, season, species and category as factors and number of animals transported per truck (NATT) as dependent variables. Results expressed as Least Square means ± standard error.
