# Supplementary material for: Road Transport of Farm Animals: Mortality, Morbidity, Species and Country of Origin at a Southern Italian Control Post
Source: Animals (Basel). 2018 Sep 17;8(9):155. doi: 10.3390/ani8090155 (PMC6162402; doi:10.3390/ani8090155)
Supplement: Supplementary file 1 [file animals-08-00155-s001.pdf]

Figure S1. Trends of the number of trucks by year and species and by month and species.

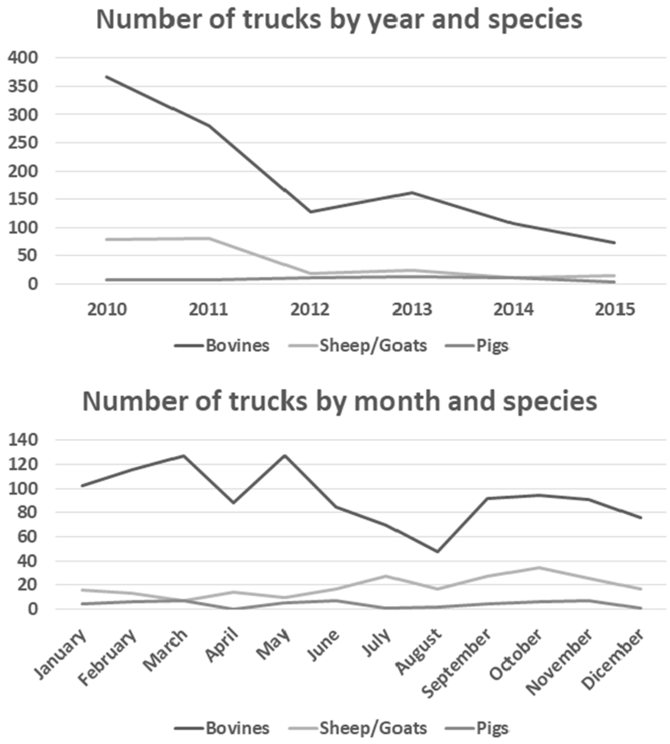

Figure S2. Association between species and nationality of Transport Company and destination.

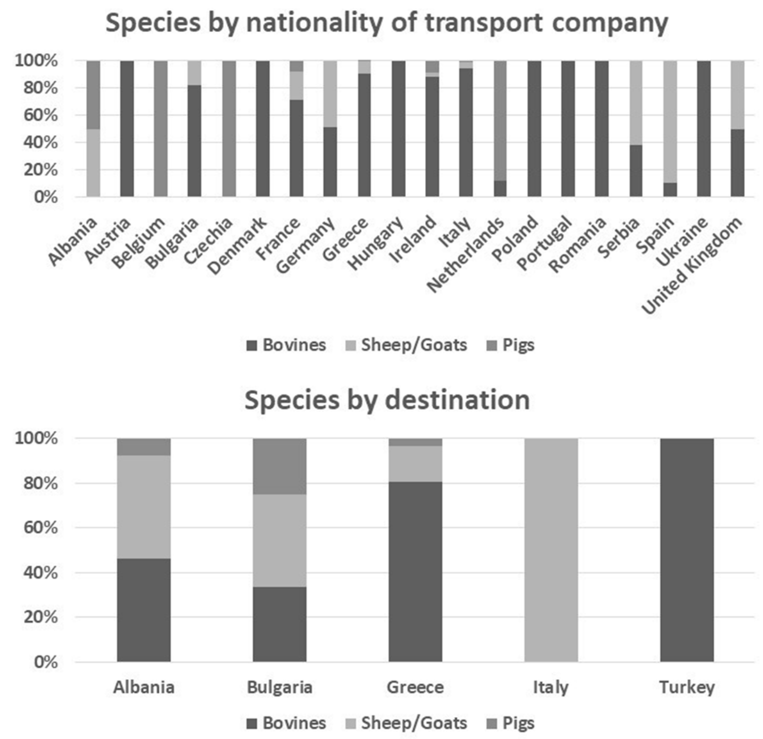

Table S1: Wald test P-values generated from univariate regression analysis

Predictive Variable	Welfare problem
Year	0.571
Month	0.998
Season	0.893
Species	0.049
Category	0.527
Provenance	1.000
Destination	0.078
Nationality	0.999
Space allowance	0.003

Table S2. Results of the GLM with year, month, season, species and category as factors and number of animals transported per truck (NATT) as dependent variables. Results expressed as Least Square means ± standard error.

**Independent Variable**	**NATT**
**P of Year**	0.072
2010	75.29 ± 4.23
2011	81.98 ± 4.69
2012	77.89 ± 7.20
2013	83.38 ± 6.41
2014	72.01 ± 7.99
2015	105.59 ± 9.43
**P of Month**	**<0.0001**
January	68.35 ± 8.06^{bc}
February	64.33 ± 7.67^{bc}
March	57.20 ± 7.50^{c}
April	80.78 ± 8.82^{abd}
May	57.78 ± 7.47^{c}
June	86.43 ± 8.53^{abd}
July	102.17 ± 8.99^{d}
August	98.48 ± 10.88^{ad}
September	89.76 ± 8.03^{d}
October	102.80 ± 7.70^{d}
November	92.04 ± 7.99^{d}
December	82.36 ± 9.19^{abd}
**P of Season**	**<0.0001**
Winter	65.26 ± 4.74^{a}
Spring	70.59 ± 4.64^{a}
Summer	96.38 ± 5.37^{b}
Autumn	91.40 ± 4.53^{b}
**P of Species**	**<0.0001**
Pigs	106.66 ± 4.75^{ab}
Sheep/goats	268.68 ± 2.24^{a}
Bovines	40.99 ± 1^{b}
**P of Category**	**<0.0001**
Sheep/goats	242.41 ± 2.07^{acdf}
Lambs	334.78 ± 3.29^{c}
Medium size calves	67.48 ± 2.26^{d}
Heavy calves	55.37 ± 2.05^{ac}
Medium size cattle	43.62 ± 2.69^{e}
Heavy cattle	32.50 ± 0.99^{b}
Very heavy cattle	21.72 ± 7.94^{f}
Light fattening	118.95 ± 4.16^{a}
Heavy fattening	70.00 ± 11.77^{g}
Breeding	45.00 ± 11.77^{d}
**P of Provenance**	**<0.0001**
Austria	33.00 ± 41.22^{ac}
Belgium	86.00 ± 35.70 ^{ac}
Denmark	20.00 ± 71.40 ^{ac}
France	58.65 ± 2.26 ^{a}
Germany	32.57 ± 26.98 ^{ac}

Hungary	80.00 ± 71.40 [abc]
Ireland	67.32 ± 7.03 [a]
Italy	37.44 ± 8.72 [c]
Luxembourg	33.00 ± 50.49 [a]
Netherlands	32.85 ± 26.98 [ac]
Portugal	75.00 ± 31.93 [ac]
Romania	38.00 ± 71.40 [a]
Spain	216.78 ± 5.11 [b]
United Kingdom	52.33 ± 41.22 [a]
**P of Destination**	**<0.0001**
Albania	114.23 ± 24.78 [ad]
Bulgaria	188.33 ± 25.79 [b]
Greece	79.19 ± 2.45 [ac]
Italy	203.00 ± 44.6 [bd]
Turkey	53.85 ± 15.10 [c]
**Nationality of transport company**	**<0.001**
Albania	94.50 ± 54.66 [abcd]
Austria	30.50 ± 54.66 [ac]
Belgium	69.80 ± 34.57 [ac]
Bulgaria	90.53 ± 8.10 [a]
Czechia	147.00 ± 77.30 [b]
Denmark	33.00 ± 54.66 [ac]
France	100.77 ± 6.97 [a]
Germany	169.92 ± 12.38 [b]
Greece	60.15 ± 3.10 [c]
Hungary	45.06 ± 13.75 [c]
Ireland	66.64 ± 7.58 [c]
Italy	79.15 ± 6.80 [c]
Netherlands	86.76 ± 15.46 [ac]
Poland	42.25 ± 11.27 [c]
Portugal	75.00 ± 34.57 [ac]
Romania	18.00 ± 44.63 [ac]
Serbia	169.47 ± 10.23 [b]
Spain	238.40 ± 9.44 [d]
Ukraine	32.03 ± 13.47 [c]
United Kingdom	30.00 ± 54.66 [ac]

Legend. In the same column, means with different superscripts differ significantly (a, b, c, d, e, f $P<0.05$).
